# pH controls spermatozoa motility in the Pacific oyster (*Crassostrea gigas*)

**DOI:** 10.1242/bio.031427

**Published:** 2018-02-26

**Authors:** Myrina Boulais, Marc Suquet, Eve Julie Arsenault-Pernet, Florent Malo, Isabelle Queau, Patricia Pignet, Dominique Ratiskol, Jacqueline Le Grand, Matthias Huber, Jacky Cosson

**Affiliations:** 1 CNRS, UMR 6539 Lemar (UBO-CNRS-IRD-Ifremer), IUEM, Plouzané 29280, France; 2 Ifremer, UMR 6539 Lemar (UBO-CNRS-IRD-Ifremer), Site expérimental d'Argenton, Landunvez 29840, France; 3 Ifremer, LMEE, Centre de Bretagne, Plouzané 29280, France; 4 University of South Bohemia in Ceske Budejovice, Faculty of Fisheries and Protection of Waters, South Bohemian Research Center of Aquaculture and Biodiversity of Hydrocenoses, Vodnany 38925, Czech Republic

**Keywords:** Seminal fluid, Spermatozoa, Motility, pH, Salinity, Ions

## Abstract

Investigating the roles of chemical factors stimulating and inhibiting sperm motility is required to understand the mechanisms of spermatozoa movement. In this study, we described the composition of the seminal fluid (osmotic pressure, pH, and ions) and investigated the roles of these factors and salinity in initiating spermatozoa movement in the Pacific oyster, *Crassostrea gigas*. The acidic pH of the gonad (5.82±0.22) maintained sperm in the quiescent stage and initiation of flagellar movement was triggered by a sudden increase of spermatozoa external pH (pHe) when released in seawater (SW). At pH 6.4, percentage of motile spermatozoa was three times higher when they were activated in SW containing 30 mM NH_4_Cl, which alkalinizes internal pH (pHi) of spermatozoa, compared to NH_4_Cl-free SW, revealing the role of pHi in triggering sperm movement. Percentage of motile spermatozoa activated in Na^+^-free artificial seawater (ASW) was highly reduced compared to ASW, suggesting that change of pHi triggering sperm motility was mediated by a Na^+^/H^+^ exchanger. Motility and swimming speed were highest in salinities between 33.8 and 42.7‰ (within a range of 0 to 50 ‰), and pH values above 7.5 (within a range of 4.5 to 9.5).

## Abstract (precis)

**Summary:** Oyster sperm motility is not triggered in an acidic environment and motility is controlled by internal pH alkalinization, mediated by a Na+/H+ exchanger.

## INTRODUCTION

Movement of spermatozoa is required for external fertilization, allowing male gametes to reach and penetrate oocytes (Billard, 1983). In invertebrates and fish, sperm is non-motile in the gonad (Cosson et al., 2008; Demoy-Schneider et al., 2014). Sperm quiescence maintains energy stores of spermatozoa and prevents alterations to their morphology (Morisawa, 1985). Initiation of sperm motility takes place after sperm release into the aquatic environment and is triggered by physical or chemical changes occurring between the gonad and the aquatic environment (Alavi and Cosson, 2005, 2006). Consequently, identifying factors inhibiting and stimulating spermatozoa movement requires the description of the chemical constituents of seminal fluid and study of their roles in controlling sperm motility.

Factors activating sperm motility vary among species. In fish, the change in osmotic pressure, pH or ion composition induces sperm movement (Morisawa and Suzuki, 1980; Alavi and Cosson, 2005, 2006). In marine invertebrates, sperm activation factors have been mainly investigated in sea urchins. Rothschild (1928) first suggested that sperm motility initiation of the sea urchin, *Echinus esculentus*, depends on the ‘dilution effect’ of spermatozoa at spawning. Later, Mohri and Yasumasu (1963) listed several factors suggested to maintain sperm quiescence in the seminal fluid, including concentrations of CO_2,_ O_2_ and ions (K^+^, Cu^2+^ or Zn^2+^), the presence of androgamone I, and pH. The crucial role of pH in triggering sperm motility was later confirmed in the sea urchin *Strongylocentrotus purpuratus*. In this species, seminal fluid pH and sperm intracellular pH (pHi) are more acidic than seawater (SW) (Schackmann et al., 1981; Christen et al., 1986). Finally, artificially increasing sperm pHi by adding NH_4_Cl to the activating medium also triggers sperm motility (Christen et al., 1982).

In marine bivalve molluscs, our knowledge of sperm biology is limited because their high fecundity compensates for the high inter-breeder variability of gamete quality, allowing mass production of marine bivalves regardless of gamete biology (Robert and Gérard, 1999). However, an understanding of the factors inhibiting and triggering the initiation of sperm motility is needed to support the current development of aquaculture that is oriented towards controlled crosses, selective breeding, and gamete cryobanking (Piferrer et al., 2009). As in sea urchins, acidic external pH (pHe) inhibits sperm movement in the Japanese pearl oyster (*Pinctada fucata martensii*; Ohta et al., 2007), black lip pearl oyster (*Pinctada margaritifera*; Demoy-Schneider et al., 2012, 2014), king scallop (*Pecten maximus*; Faure et al., 1994), and Pacific oyster (*Crassostrea gigas*; Dong et al., 2002; Alavi et al., 2014). A complex interaction between environmental pH and temperature modified sperm movement characteristics in the mussel, *Mytilus galloprovincialis* (Eads et al., 2016). Recently, it was shown that the concentration of some gonadal ions (i.e. K^+^, Ca^2+^, and Na^+^) are involved in controlling sperm motility in the Pacific oyster (Alavi et al., 2014). However, the roles of pHi, other ions of the seminal fluid such as Mg^2+^, the osmotic pressure, and salinity in triggering motility initiation have never been investigated in Pacific oyster spermatozoa. Furthermore, control of swimming speed, a key feature of sperm motility, by chemical factors stimulating and inhibiting motility has never been studied in bivalve species.

In this study, we described the composition of the seminal fluid (pH, ions, and osmotic pressure) and investigated the roles of these factors and salinity in initiating spermatozoa movement (percentage of motility and swimming speed) in the Pacific oyster, a model species in invertebrate reproduction.

## RESULTS

### Composition of the seminal fluid

Osmotic pressure, pH, and concentrations of ions of Pacific oyster seminal fluid are reported in Table 1. Seminal fluid pH of the Pacific oyster was acidic. Two ions, Cl^−^ and Na^+^, were predominant in the seminal fluid.

**Table 1.**
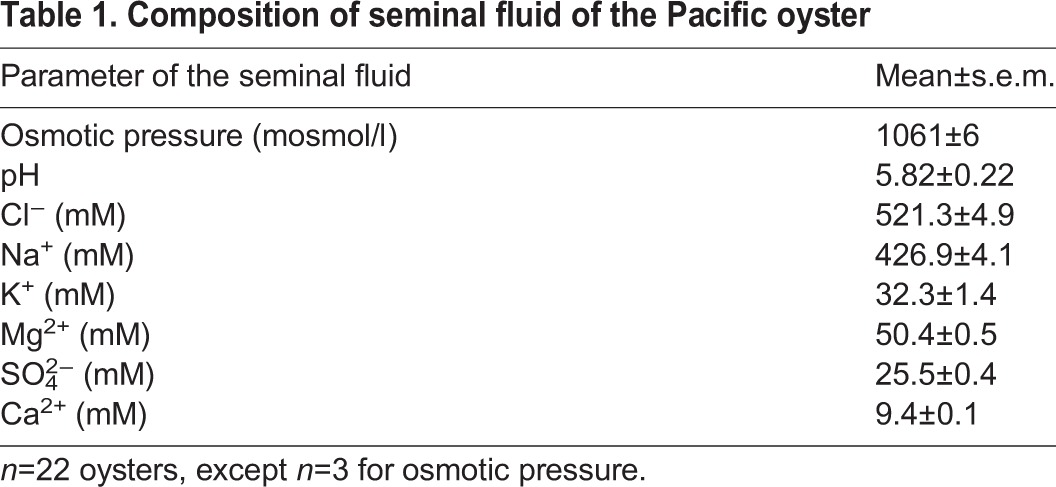
**Composition of seminal fluid of the Pacific oyster**

### Effects of salinity, pH, and ions in initiating spermatozoa movement

**Fig. 1.**
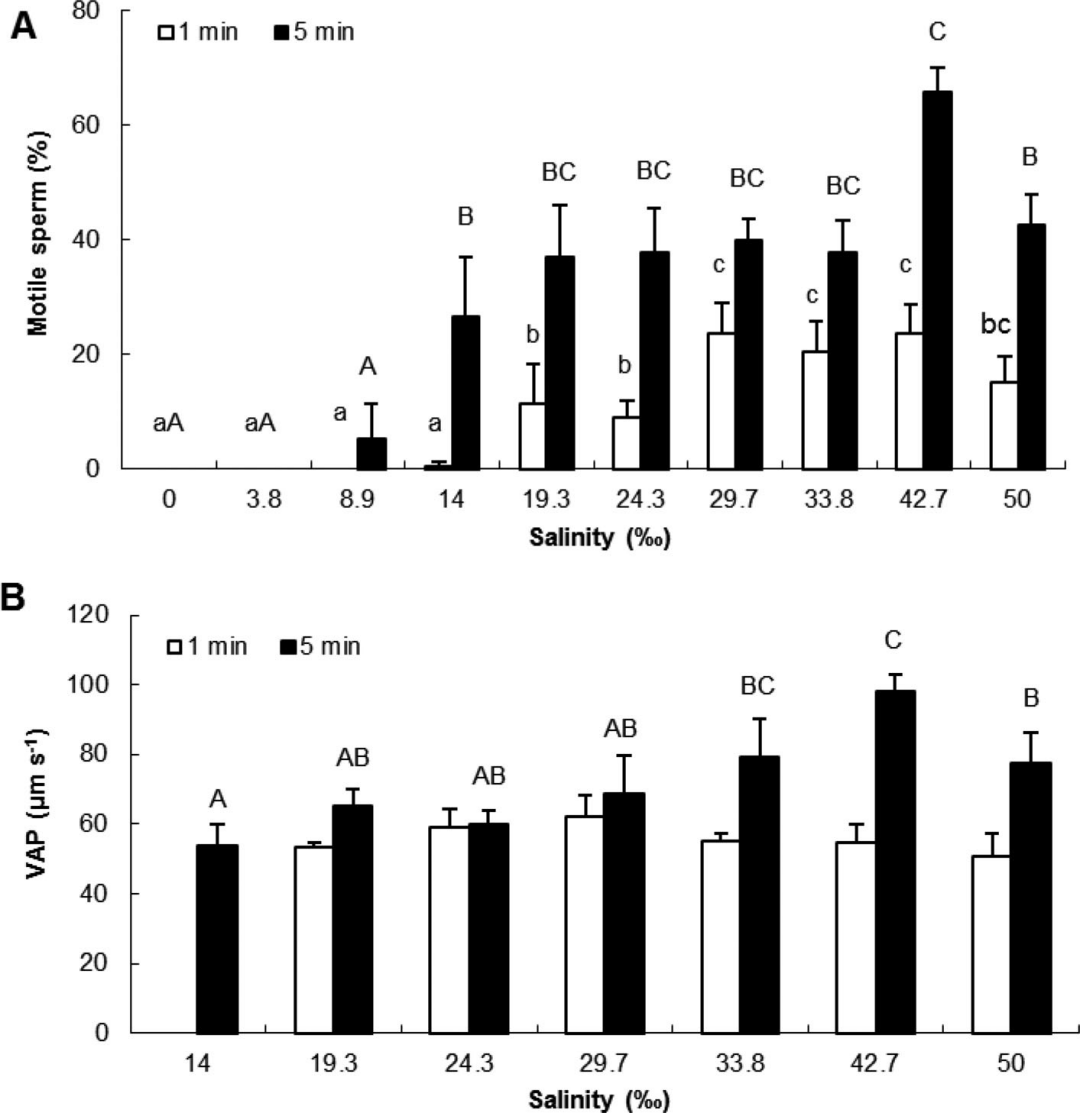
**Effects of salinity on percentage of motile spermatozoa and VAP after 1 and 5 min after activation.** Motile spermatozoa (A, *P*=0.000), VAP (B, *P=*0.003); VAP, Velocity of the Average Path. VAP in salinities of 3.9 ‰, 8.9 ‰ (1 and 5 min after activation), and 14 ‰ (1 min after activation) was not measured because of low number of motile spermatozoa. VAP was not significantly different among salinities for spermatozoa activated for 1 min. Different letters refer to significantly different results (mean±s.e.m.; lower case letters, 1 min after sperm activation; upper case letters, 5 min after sperm activation; *n*=5 oysters). Data were compared using one-way ANOVA and Fisher *a posteriori* test.

The percentage of motile spermatozoa significantly increased with salinity both at 1 min after activation (*F_1,9_*=15.88, *P*<0.001) and 5 min after activation (*F_1,9_*=12.11, *P*<0.001) (Fig. 1A). At 5 min post activation, the highest values were recorded for salinity ranging from 19.3 to 42.7‰. Velocity of the Average Path (VAP) was not significantly different among salinities for spermatozoa activated for 1 min. However, VAP significantly increased (*F_1,7_*=4.06, *P<*0.01) with salinity for spermatozoa activated for 5 min, maximum VAP values being recorded at 33.8 and 42.7‰ (Fig. 1B). Overall, there was a trend for highest motility and VAP in salinities between 33.8 and 42.7‰.

**Fig. 2.**
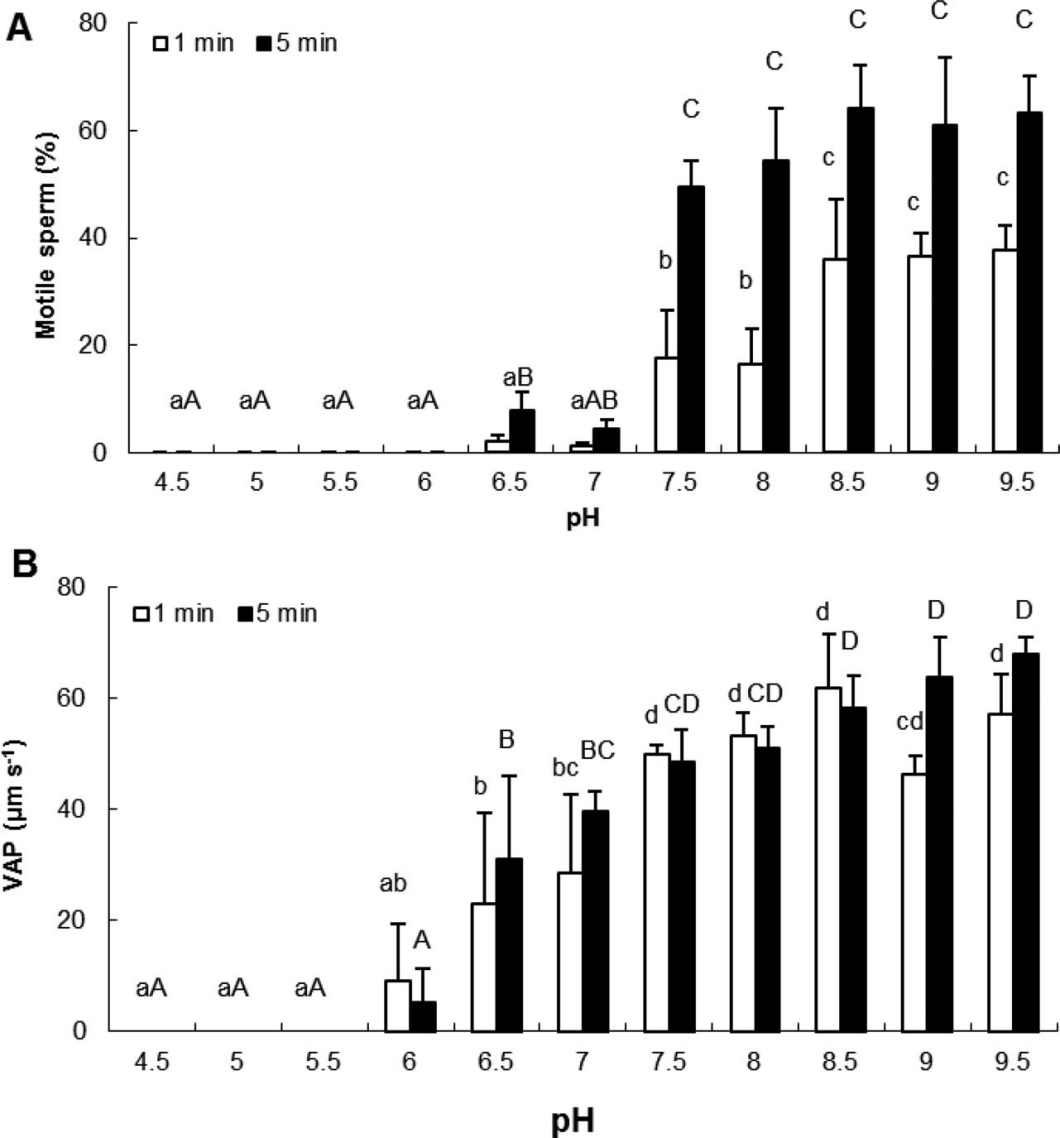
**Effects of pH on percentage of motile spermatozoa and VAP after 1 and 5 min after activation.** Motile spermatozoa (A, *P*=0.000), VAP (B, *P*=0.000); VAP, Velocity of the Average Path. Different letters refer to significantly different results (mean±s.e.m.; lower case letters, 1 min after sperm activation; upper case letters, 5 min after sperm activation; *n*=5 oysters). Data were compared using one-way ANOVA and Fisher *a posteriori* test.

Oyster sperm was non-motile in pH values ranging from 4.5 to 5.5 (Fig. 2A). Low percentages of motile spermatozoa (<10%) were recorded at pH 6.0, 6.5, and 7.0. A significant increase of motile percentage was found at pH values of 7.5 and above, both at 1 min after activation (*F_1,10_*=20.71, *P*<0.001) and 5 min after activation (*F_1,10_*=44.94, *P*<0.001). For spermatozoa activated for 1 min, the percentage of motile cells significantly increased between pH 8.0 and 8.5, which was not the case for spermatozoa activated for 5 min. Sperm VAP significantly increased in a pH-dependent manner from pH 6.5 for both sperm movement durations (*F_1,10_*=11.26 at 1 min and *F_1,10_*=24.31 at 5 min, *P*<0.001 for both durations) (Fig. 2B).

**Fig. 3.**
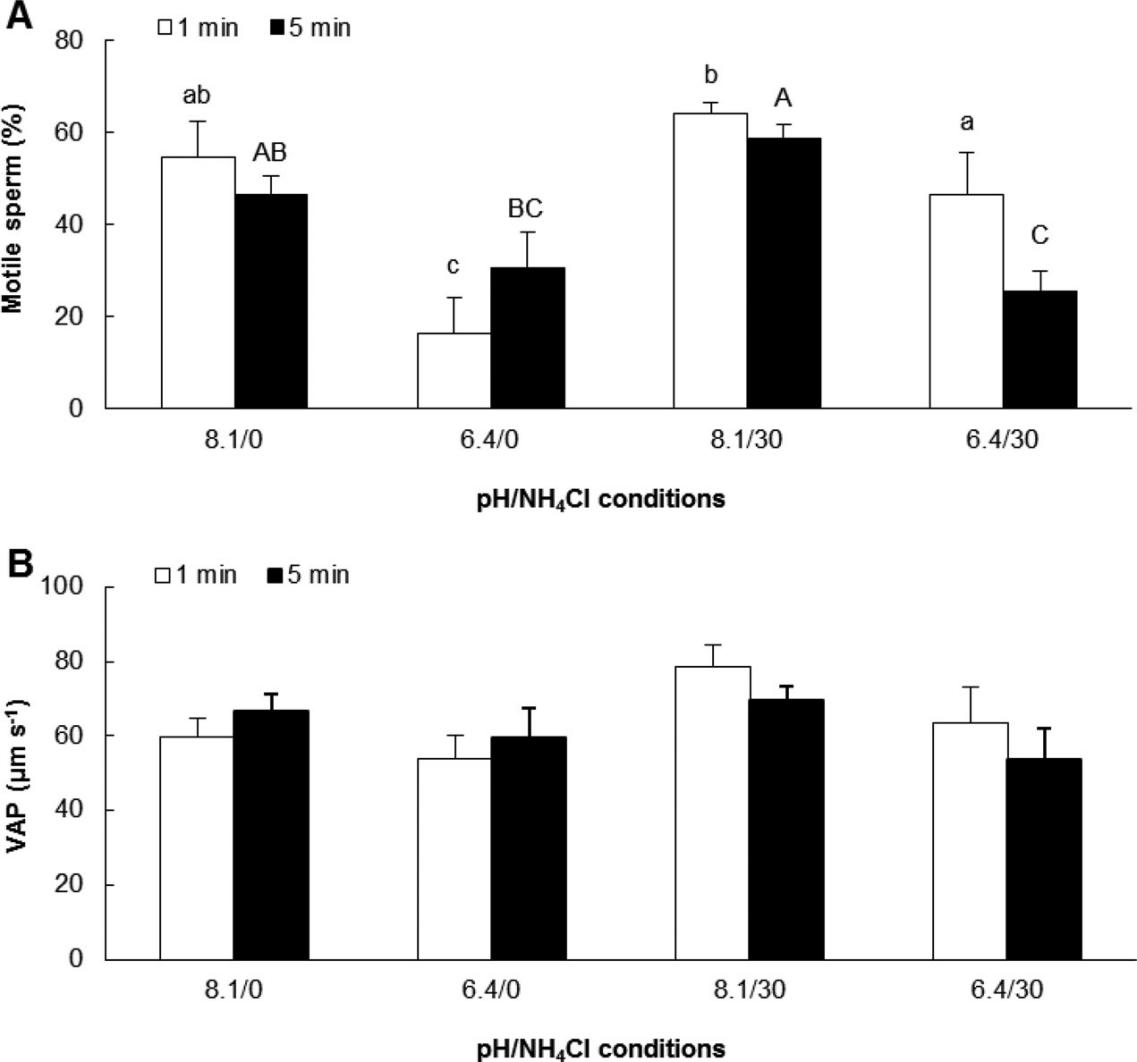
**Effect of NH_4_Cl (0 or 30 mM) on percentage of motile spermatozoa and VAP after 1 and 5 min after activation.** Motile spermatozoa (A, *P*=0.000), VAP (B); VAP, Velocity of the Average Path. VAP was not significantly different for both NH_4_Cl concentrations (B). Different letters refer to significantly different results (mean±s.e.m.; lower case letters, 1 min after sperm activation; upper case letters, 5 min after sperm activation; *n*=5 oysters). Data were compared using one-way ANOVA and Fisher *a posteriori* test.

Regarding the effect of increasing spermatozoa pHi by adding NH_4_Cl to the activating media, the percentage of motile spermatozoa at 1 min after activation was significantly lower at pH 6.4 compared to pH 8.1 when NH_4_Cl was not added (*F_1,5_*=18.71, *P*<0.001) (Fig. 3A). NH_4_Cl (30 mM) significantly increased the percentage of motile spermatozoa activated in pH 6.4 for 1 min. The percentage of motility after 5 min of sperm activation was not significantly changed by NH_4_Cl compared to controls (i.e. NH_4_Cl-free SW for each corresponding pH). VAP was similar for both NH_4_Cl concentrations (Fig. 3B).

**Fig. 4.**
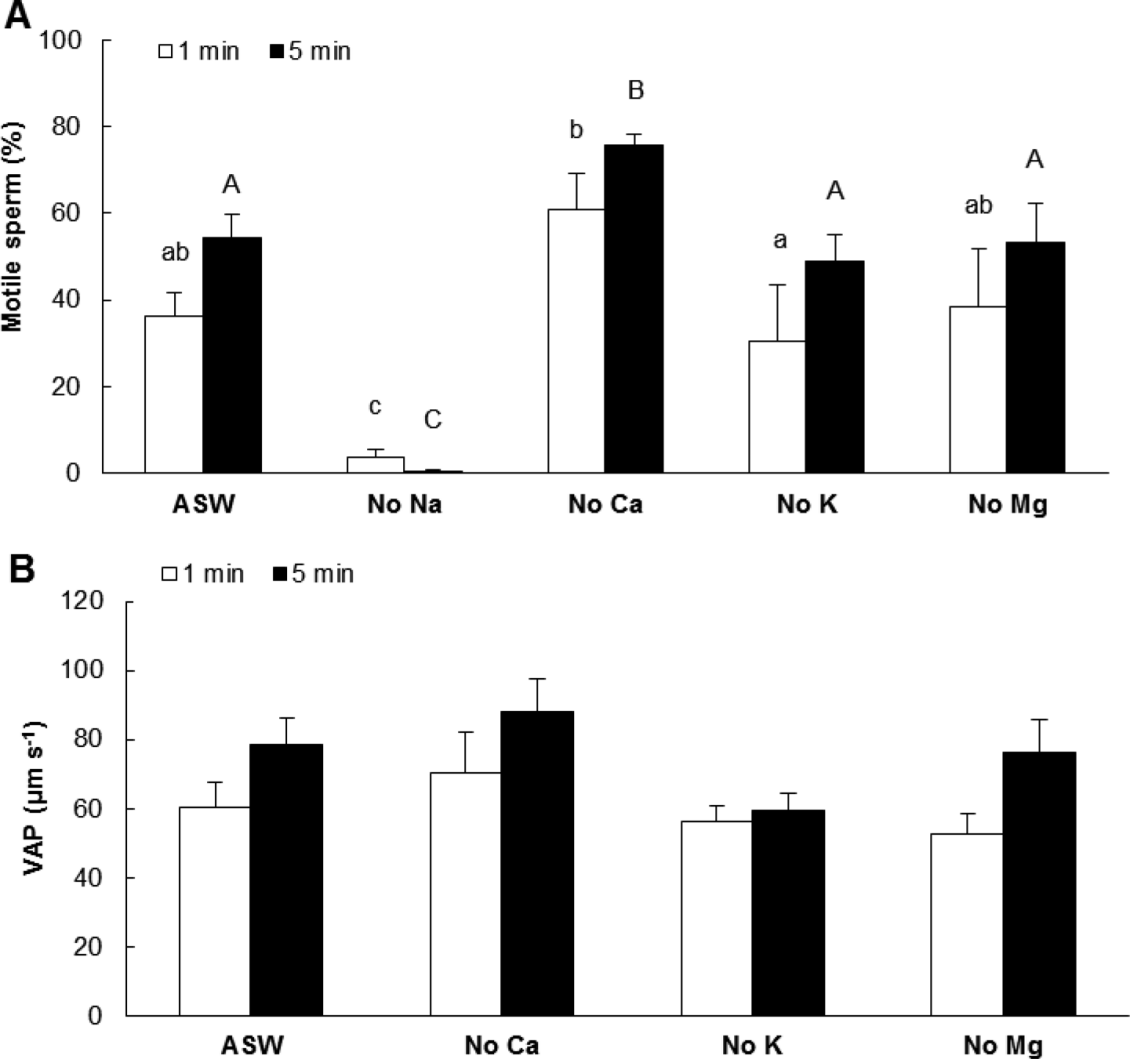
**Effects of ion deprivation on percentage of motile spermatozoa and VAP after 1 and 5 min after activation.** Motile spermatozoa (A, *P*=0.006 at 1 min and *P*=0.000 at 5 min after activation), VAP (B); VAP, Velocity of the Average Path. VAP in Na^+^-free ASW was not measured because of low number of motile spermatozoa. No movement of spermatozoa was observed in ion-free ASW. Sperm VAP was not significantly affected by a lack of Ca^2+^, K^+^ or Mg^2+^ in ASW. ASW, artificial seawater; different letters refer to significantly different results (mean±s.e.m.; lower case letters, 1 min after sperm activation; upper case letters, 5 min after sperm activation; *n*=5 oysters). Data were compared using one-way ANOVA and Fisher *a posteriori* test.

VAP and percentage of motile spermatozoa were not significantly different between SW and artificial seawater (ASW) regardless of the activation time (Fig. S1). Consequently, ASW was considered as the single control for the following results. At both times after activation, a significantly lower percentage of motility (*F_1,5_*=4.37, *P*<0.01 at 1 min and *F_1,5_*=44.85, *P*<0.001 at 5 min after activation) was recorded for Na^+^-free media compared to ASW (Fig. 4A). Conversely, a lack of Ca^2+^ significantly increased the percentage of motile spermatozoa activated for 5 min, but not for 1 min, compared to ASW. A lack of K^+^ or Mg^2+^ in activating medium did not significantly change percentage of motile spermatozoa. Sperm VAP was not significantly affected by a lack of Ca^2+^, K^+^ or Mg^2+^ in ASW (Fig. 4B, VAP in Na^+^-free ASW was not measured because of the low number of motile spermatozoa). No movement of spermatozoa was observed in ion-free ASW.

## DISCUSSION

### The present study investigated the roles of pH, ions, and salinity in initiating Pacific oyster spermatozoa movement

Motility of spermatozoa was triggered by a sudden alkalinization of sperm environment when released in seawater. Values of pH of 7.5 and above were optimal to initiate movement of Pacific oyster spermatozoa. This value is lower than the one suggested by Alavi et al. (2014; pHe>8) in the same species. This is probably explained by the use of pH-indicator strips by these authors, a methodology which is not as accurate as a pH meter. Although this 0.5 difference in optimal pH value between our study and the one of Alavi et al. (2014) appears small, it represents very different acidic conditions due to the logarithmic nature of the pH scale. Inhibition of sperm motility in neutral and acidic pH conditions (≤7.0) demonstrated that the low pH of the gonadal environment (pH 5.82) maintains sperm in the quiescent stage. The inhibitory role of acidic pH in sperm motility was also suggested in the black-lip pearl oyster, characterized by an optimal range of pH values for sperm motility ranging from 8.5 to 10.5 (Demoy-Schneider et al., 2012). In the king scallop, pHe also regulates sperm motility initiation (Faure et al., 1994).

Since pHe mediates pHi alkalinization in spermatozoa (Christen et al., 1982), we investigated the role of pHi changes in triggering motility initiation in Pacific oyster spermatozoa. A threefold higher percentage of motile spermatozoa was observed when pHi was alkalinized using NH_4_Cl compared to NH_4_Cl-free SW. Initiation of sperm motility by alkalinization of pHi using NH_4_Cl or NH_3_ has been reported in the Manila clam (Alavi et al., 2014), sea urchin species (Christen et al., 1982, 1983; Lee et al., 1983), and starfish *Asterina pectinifera* (Nakajima et al., 2005). Initiation of sperm motility by pHi alkalinization is related to functionality of flagellar dynein ATPases, the enzymes sustaining axonemal movement. It was demonstrated that optimal pH values for dynein ATPase reactions ranged from 7.4 to 8.6, while their activity is inhibited at pH 7.2 (Gibbons and Gibbons, 1972; Christen et al., 1982).

Osmotic pressure of the seminal fluid was similar to the one of seawater (∼1000 mOsm kg^−1^ for salinity of 35 ‰; Lovett et al., 2006), demonstrating that initiation of sperm movement is osmolarity independent in the Pacific oyster. The roles of seminal fluid ions in controlling sperm movement were explored in our work. Similarly to our results, Alavi et al. (2014) recorded no movement in ion-free ASW, demonstrating that the presence of ions is required to trigger sperm movement in this species.

Movement of Pacific oyster spermatozoa was dependent on the ionic composition of the activating medium. Sperm activation in Na^+^-free ASW decreased the percentage of motility down to 1% of the value observed in the control, showing that Na^+^ plays a major role in Pacific oyster spermatozoa movement. Dilution of spermatozoa in Na^+^-free ASW also inhibited sperm motility in the king scallop (Faure et al., 1994). In sea urchins, sperm movement is triggered by a Na^+^-dependent pHi rise suggested to be mediated by a Na^+^/H^+^ exchanger (Gatti and Christen, 1985; Darszon et al., 1999). Consequently, pHi but not Na^+^ is the primary factor controlling sperm motility in sea urchins. This statement is sustained by the initiation of spermatozoa movement following pHi alkalinization using NH_4_Cl (Christen et al., 1983; Lee et al., 1983). Based on the present results, a similar mechanism is suggested in Pacific oyster spermatozoa and would be of interest to address in the future.

Our results showed a higher percentage of motile spermatozoa in Ca^2+^-free ASW compared to ASW at 5 min after activation. It was previously reported that dilution of sperm in a medium lacking Ca^2+^ increased motility of Eastern oyster and Pacific oyster spermatozoa (Paniagua-Chavez et al., 1998; Dong et al., 2002). On the other hand, a lower percentage of motile spermatozoa was observed in Ca^2+^-free ASW compared to ASW in the Pacific oyster (Alavi et al., 2014). However, these authors highlighted that intracellular Ca^2+^ concentration was not the primary factor controlling motility because the addition of Ca^2+^ ionophore to sucrose- or to Na^+^ -activating medium containing 10 mM Ca^2+^ did not trigger sperm motility. Plasma membrane Na^+^/Ca^2+^ exchangers are one way in which cells regulate Ca^2+^ (Blaustein and Lederer, 1999). We suggest that the higher percentage of motile spermatozoa in Ca^2+^-free ASW compared to ASW, observed in our study, is associated with changes in internal Na^+^ concentration. Investigating the roles of Na^+^/Ca^2+^ exchangers in controlling sperm motility initiation would be of interest for future studies.

In the present study, a lack of K^+^ in the activating medium did not significantly change sperm movement characteristics of the Pacific oyster, validating that K^+^ influx is not required to trigger sperm motility (Alavi et al., 2014). These authors suggested that K^+^ efflux, however, is essential for initiation of sperm motility and that high concentration of K^+^ in the gonad maintains sperm in the quiescent state in this species. In sea urchins, in the presence of sodium, addition of K^+^ induces an internal alkalinization, accompanied by an influx of potassium (Gatti and Christen, 1985).

Some differences in pH and ionic composition of seminal fluid are observed among marine invertebrates (Table 2). Spermatozoa activation is an evolutionary conserved mechanism which involves a Na^+^-dependent alkalinization of pHi in marine invertebrates. In marine fish, low osmotic pressure (306 to 400 mosmol l^−1^) and concentration of K^+^ (2.0 to 5.3 mmol l^−1^), and high pH value (7.3 to 7.8) of the seminal fluid (reviewed in Suquet et al., 1994) reflect a different mechanism of spermatozoa activation with respect to marine invertebrates. In marine fish, pH is not involved in triggering sperm motility, but a rise of the osmotic pressure during sperm release initiates spermatozoa movement (Morisawa and Suzuki, 1980; Cosson et al., 2008).

**Table 2.**
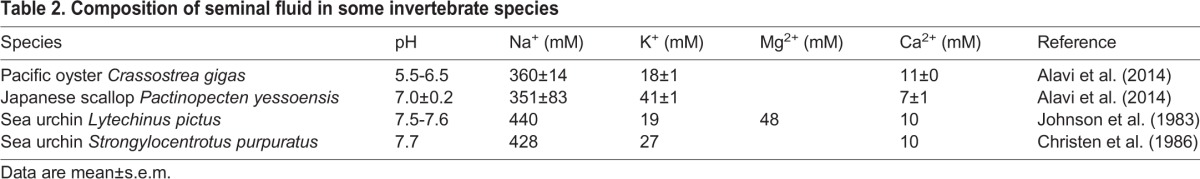
**Composition of seminal fluid in some invertebrate species**

Our results revealed that spermatozoa are equally motile in a wide range of salinities (19.3 to 42.7‰). The limited role of this environmental factor in controlling sperm motility is in agreement with optimal salinity requirements of gametogenesis and early life stage development of the Pacific oyster, ranging from 18 to 30‰ (Phelps and Warner, 1990; Helm, 2005). It was suggested that tolerance of Pacific oyster to low salinities could be sustained by molecular mechanisms of osmoadaptation (Zhao et al., 2012).

Current models predicting the degree of ocean acidification call for a drop in pH by 0.3 to 0.5 by 2100, and possibly by 0.8 to 1.4 by 2300 depending on the CO_2_ emission scenario (Caldeira and Wickett, 2005; IPCC, 2013). In the present study, decreasing SW pH from 8.0 to 7.5, as predicted by the end of this century, affected neither percentage of motile spermatozoa nor swimming speed, suggesting that near-future levels of ocean acidification are not a major threat to spermatozoa motility in the Pacific oyster. Species-specific responses are found in studies investigating the effect of low pH on spermatozoa movement of marine invertebrates. In the mussel, *Mytilus galloprovincialis*, decreasing seawater pH from 8.0 to 7.6 induced a 25% reduction of percentage of motile spermatozoa and their sperm swimming speed (Vihtakari et al., 2013). In the same species, Eads et al. (2016) found that the effect of pH on spermatozoa movement was only noticeable after 2 h of exposure. In the sea urchin, *Heliocidaris erythrogramma*, seawater acidification Δ pH of 0.3 and 0.5 units resulted in 7 and 9% reduction of percentage of motile spermatozoa, respectively, without affecting sperm swimming speed (Schlegel et al., 2012). For higher levels of ocean acidification such as predicted after 2100 (<pH 7.5), the movement characteristics of Pacific oyster spermatozoa will be adversely impacted, which may have profound negative implications for reproduction and subsequent recruitment in this species. Overall, oyster reproduction seems to be resilient to moderate environmental acidification. In the present study, acidification of sperm environment was obtained by manipulating pH by acid while best practice methodologies manipulate pH either by aerating or suturing seawater with CO_2_ (Ross et al., 2011). Investigating the effects of ocean acidification on Pacific oyster sperm movement must be further studied, taking into account acidification methodology.

In conclusion, acidic pH of the seminal fluid compared to seawater maintains sperm in the quiescent state in the gonad of the Pacific oyster. Sperm movement is suggested to be triggered by a sudden Na^+^-dependent alkalinization of pHi as a consequence of sperm release in seawater. Other cations studied in the present study on sperm motility initiation have only minor effects. The use of sperm swimming speed as a proxy of environmental conditions strengthens previous observations carried out only using the percentage of motile spermatozoa. Finally, the present observations highlight the resilience Pacific oyster sperm motility exhibits in response to ocean acidification projected for the near-future.

## MATERIALS AND METHODS

### Animals and sperm collection

Three-year-old Pacific oysters (mean weight±s.e.m., 136±11 g) were collected from aquaculture stock in the Bay of Brest (Finistère, France) during their natural spawning period. They were transferred to the Argenton experimental hatchery and maintained in conditioning conditions in a 600-l tank (natural SW at 19°C, oysters fed *ad libitum* with a mixture of two microalgae, *Isochrysis galbana* and *Chaetoceros gracilis*, equal to 8% dry weight algae/dry weight oyster per day and per oyster) until their use (Fabioux et al., 2005). Since our study was aimed at studying spermatozoa biological characteristics of ripe oysters, only stage 3 males were selected (Steele and Mulcahy, 1999). For each oyster, sperm was collected by sampling (700 µl) in the proximal part of the gonad using a micropipette, and each sperm sample was stored on ice in a 2-ml plastic vial (Eppendorf Quality™) until use.

### Composition of the seminal fluid

To determine pH, osmotic pressure and concentrations of ions of the seminal fluid (*n*=22 oysters, except *n*=3 oysters for osmotic pressure), a 500-µl subsample of sperm was centrifuged (Sigma-Aldrich 3-16PK) at 4000 ***g*** for 1 h at 4°C (1.5-ml plastic vial Eppendorf Quality™) for each individual sperm sample. Supernatant (i.e. seminal fluid) pH was immediately assessed using a pH meter (IQ150, IQ Scientific instrument; total pH scale). The supernatant was then collected in a 500-µl plastic vial (Eppendorf Quality™) and stored at −28°C until later analyses of osmotic pressure and concentrations of ions. Seminal fluid osmotic pressure was measured on a 100-µl subsample, using an automatic osmometer (Type 15, Löser Messtechnik, Berlin, Germany). Concentrations of ions were assessed using ion chromatography (50 µl subsample, ICS 900, Thermo Fisher Scientific) fitted with a Ionpac CS 12A column and a suppressor (CSRS-ultra II) unit in combination with a DS5 conductivity cell (Thermo Fisher Scientific). Components were separated using a methasulfonic acid eluent (18 mM), with a flow of 1 ml min^−1^ (Lazar et al., 2011).

### Effects of salinity, pH, and ions in initiating spermatozoa movement

To study the effects of salinity, pH and ions on triggering movement, 5 µl of sperm was diluted in 500 µl of different activating media described below (500-µl plastic vial, Eppendorf Quality™) for each oyster (*n*=5 oysters). Unless stated, activating media consisted of SW filtered at 1 µm, 34.5‰ salinity, 19°C, supplemented by 0.3% pluronic acid, 15 mM Tris, and final pH adjusted to 8.1 by addition of diluted HCl (all chemicals used in our experiments were purchased from Sigma-Aldrich^®^).

The effects of salinity on sperm movement activation were explored across a range of salinities, from 0.4 to 50‰. Adjustment of salinity was conducted by dilution of activating media with distilled water or by addition of NaCl solution for salinity values higher than 35‰. The effects of pHe on sperm movement initiation was tested after adjusting pH of activating media to values ranging from 4.5 to 9.5. Buffers used to adjust pH values were 15 mM acetate/acetic acid and 15 mM Tris/HCl for pH values between 4.5 and 6.0 and between 6.5 and 9.5, respectively. The effect of spermatozoa pHi alkalinization on their movement characteristics was tested at two different pH values (6.4 and 8.1), adding NH_4_Cl (30 mM final) as this chemical alkalinizes pHi (Christen et al., 1983).

Finally, to explore the effects of seawater ions on triggering sperm motility, different activating media were prepared, including artificial seawater (ASW) and Na^+^-, Ca^2+^-, K^+^-, Mg^2+^-, and ion-free ASW (Table 3). Ethylene glycol-bis(β-aminoethyl ether)-N,N,N’,N'-tetraacetic acid (EGTA) was used in the ion free ASW medium because sugar may be contaminated by trace concentration of Ca^2+^. EGTA and ethylenediaminetetraacetic acid (EDTA) had no toxic effects on sperm motility at concentration of 1 mM as these chemicals did not decrease sperm movement characteristics in Ca^2+^- and Mg^2+^-free ASW.

**Table 3.**
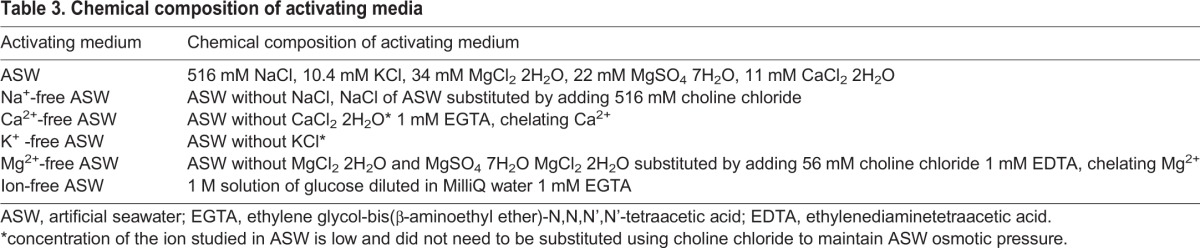
**Chemical composition of activating media**

### General procedure for assessment of sperm motility

After sperm dilution in the activating medium, a 12 µl sample was transferred to a Fast read 102 cell (Biosigma, Cona, Italy). Sperm movement was recorded at the top of the drop, and at 1 and 5 min after dilution in activating media, with maximum values of sperm movement being previously observed after 5 min of activation (Suquet et al., 2016). Sperm samples were observed using a phase contrast microscope (Olympus BX51, ×20 magnification) connected to a video camera (Qicam Fast 1934, 60 frames s^−1^ camera). Percentage of motile spermatozoa and their VAP were assessed (*n*>30 spermatozoa for each assay) using a computer-assisted sperm analyser (CASA) plugin developed for the ImageJ software (Wilson-Leedy and Ingermann, 2007) and calibrated to Pacific oyster according to Boulais et al. (2015).

### Statistical analysis

Data were presented as mean±s.e.m. Percentage data were arcsin square-root transformed. Normality (Shapiro-Wilk test) and homogeneity of variances (Levene's test) were verified. Data were compared using one-way ANOVA. When significant differences were observed, a Fisher *a posteriori* test was used. Experimental results were considered significant at *P*<0.05. Statistical analyses were performed using Statistica 6 software (StatSoft).

## Supplementary Material

Supplementary information
