# Supplementary material for: pH controls spermatozoa motility in the Pacific oyster (Crassostrea gigas)
Source: Biol Open. 2018 Feb 26;7(3):bio031427. doi: 10.1242/bio.031427 (PMC5898264; doi:10.1242/bio.031427)
Supplement: Supplementary information [file biolopen-7-031427-s1.pdf]

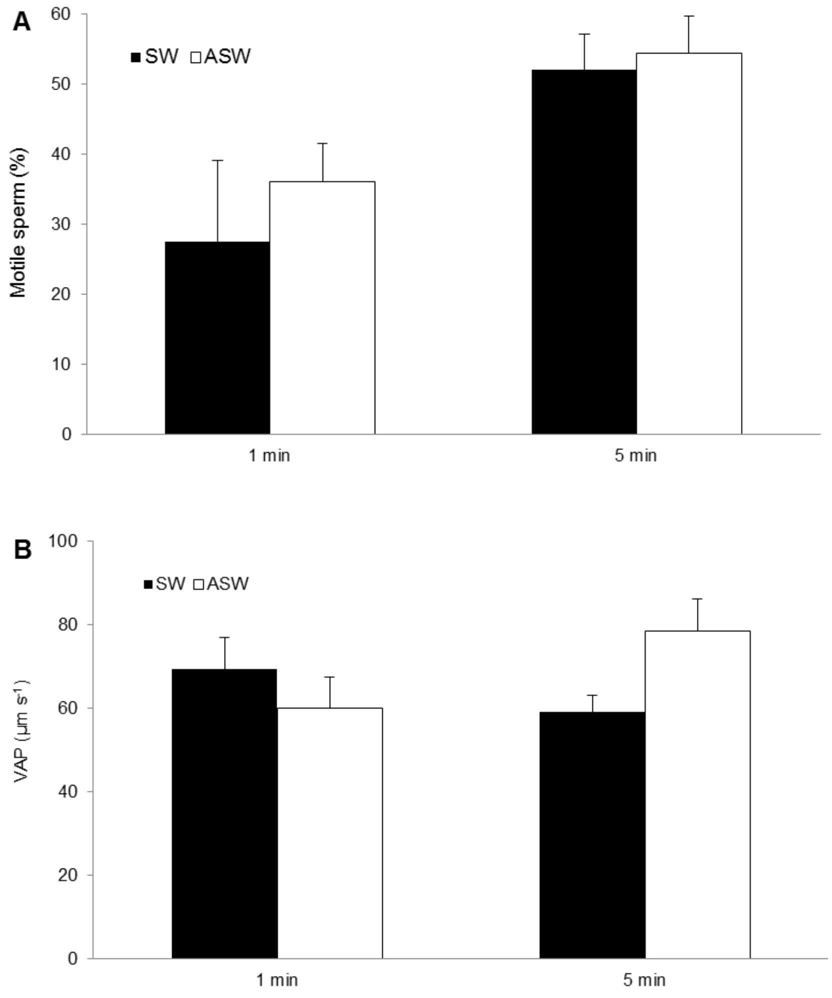

Fig. S1. Percentage of motile spermatozoa and VAP in seawater and artificial seawater after 1 and 5 min after activation. Motile (A), VAP (B), VAP: Velocity of the Average Path, SW: seawater, ASW: artificial seawater, mean ± SEM, n = 5 oysters.
